# Oxidative Stress and Inflammation: What Polyphenols Can Do for Us?

**DOI:** 10.1155/2016/7432797

**Published:** 2016-09-22

**Authors:** Tarique Hussain, Bie Tan, Yulong Yin, Francois Blachier, Myrlene C. B. Tossou, Najma Rahu

**Affiliations:** ^1^Key Laboratory of Agroecological Processes in Subtropical Region, Institute of Subtropical Agriculture, Chinese Academy of Sciences, Observation and Experiment Station of Animal Nutrition and Feed Science in South-Central China, Ministry of Agriculture, Hunan Provincial Engineering Research Center for Healthy Livestock and Poultry Production, Changsha, Hunan 410125, China; ^2^University of the Chinese Academy of Sciences, Beijing 10008, China; ^3^Hunan Collaborative Innovation Center for Utilization of Botanical Functional Ingredients and Hunan Collaborative Innovation Center of Animal Production Safety, Changsha, Hunan 410000, China; ^4^UMR 914 INRA/Agro Paris Tech, Nutrition Physiology and Ingestive Behavior, Paris, France; ^5^Department of Veterinary Microbiology, Faculty of Animal Husbandry and Veterinary Sciences, Sindh Agriculture University, Tandojam, Sindh 70050, Pakistan

## Abstract

Oxidative stress is viewed as an imbalance between the production of reactive oxygen species (ROS) and their elimination by protective mechanisms, which can lead to chronic inflammation. Oxidative stress can activate a variety of transcription factors, which lead to the differential expression of some genes involved in inflammatory pathways. The inflammation triggered by oxidative stress is the cause of many chronic diseases. Polyphenols have been proposed to be useful as adjuvant therapy for their potential anti-inflammatory effect, associated with antioxidant activity, and inhibition of enzymes involved in the production of eicosanoids. This review aims at exploring the properties of polyphenols in anti-inflammation and oxidation and the mechanisms of polyphenols inhibiting molecular signaling pathways which are activated by oxidative stress, as well as the possible roles of polyphenols in inflammation-mediated chronic disorders. Such data can be helpful for the development of future antioxidant therapeutics and new anti-inflammatory drugs.

## 1. Introduction

Oxidative stress refers to the excessive production of reactive oxygen species (ROS) in the cells and tissues and antioxidant system cannot be able to neutralize them. Imbalance in this protective mechanism can lead to the damage of cellular molecules such as DNA, proteins, and lipids [1]. Reactive oxygen species are normally produced within the body in limited quantity and are important compounds involved in the regulation of processes involving the maintaining of cell homeostasis and functions such as signal transduction, gene expression, and activation of receptors [2]. Mitochondrial oxidative metabolism in cells produces ROS species and organic peroxides in the process of cell respiration [3]. In addition, in hypoxic conditions, nitric oxide may also be produced during the respiratory chain reaction [4]. This latter reactive nitrogen species (RNS) may further lead to the production of reactive species such as reactive aldehydes, malondioaldehyde, and 4-hydroxynonenal [5]. Main targets of oxidative stress are proteins, lipids, and DNA/RNA, and modifications in these molecules may increase the chances of mutagenesis. ROS/RNS overproduction notably over a prolonged period of time can cause damage of the cellular structure and functions and may induce somatic mutations and preneoplastic and neoplastic transformations. Then, excessive production of ROS in cells and tissues may be deleterious if not removed quickly [6]. Indeed, excessive ROS/RNS production may cause irreversible damage to cells resulting in cell death by the necrotic and apoptotic processes [7].

Polyphenols are natural compounds present in plants with numerous biological activities. Phenolic compounds and flavonoids can interact with ROS/RNS and thus terminate chain reaction before cell viability is seriously affected [21].

Various inflammatory stimuli such as excessive ROS/RNS produced in the process of oxidative metabolism and some natural or artificial chemicals have been reported to initiate the inflammatory process resulting in synthesis and secretion of proinflammatory cytokines. Activation of nuclear factor-kappa B/active protein-1 (NF-*κ*B/AP-1) and production of tumor necrosis factor-alpha (TNF-*α*) have been for instance documented to play critical role in the inflammatory process resulting in several chronic diseases. Phytochemicals such as polyphenols have been reported to be able to modulate the inflammatory processes [22].

This review paper was designed to highlight the biological effects of the polyphenols and their potential to act as compounds with anti-inflammatory properties.

## 2. Relationships between Oxidative Stress and Inflammation

Inflammation is a natural defense mechanism against pathogens and it is associated with many pathogenic diseases such as microbial and viral infections, exposure to allergens, radiation and toxic chemicals, autoimmune and chronic diseases, obesity, consumption of alcohol, tobacco use, and a high-calorie diet. Many of chronic diseases linked with higher production of ROS result in oxidative stress and variety of protein oxidations [23]. Furthermore, protein oxidations turn into release of inflammatory signals molecules and peroxiredoxin 2 (PRDX2) has been recognized as an inflammatory signal [24].

Relationship between oxidative stress and inflammation has been documented by many authors. Evidences indicated that oxidative stress plays a pathogenic role in chronic inflammatory diseases. Damage of oxidative stress such as oxidized proteins, glycated products, and lipid peroxidation results in neuron degenerations mostly reported in brain disorders [25]. ROS generated in brain tissues can modulate synaptic and nonsynaptic communication between neurons that result in neuroinflammation and cell death and then in neurodegeneration and memory loss [25].

Tripeptide glutathione (GSH) is an intracellular thiol antioxidant; lower level of this GSH causes higher ROS production, which results in imbalanced immune response, inflammation, and susceptibility to infection [26]. Study was conducted on the role of GSH and its oxidized form and their regulatory function and gene expressions beyond free radical scavenging activities linked with GSH. GSH takes part in the redox regulation of immunity [27] through mixed disulfides between protein cysteines and glutathiones; it is known as glutathionylation which operates signaling proteins and transcription factors [28].

Inflammatory stimuli induce the release of PRDX2, a ubiquitous redox-active intracellular enzyme. After releasing, it acts as a redox dependent inflammatory mediator and activates macrophages to produce and release TNF-*α*. Oxidative linked GSH to PRDX2 protein glutathionylation occurs before or during PRDX2 release which regulates immunity. Salzano et al. identified PRDX2 among the glutathionylated proteins released in vitro by LPS-stimulated macrophages using mass spectrometry proteomic methods [24]. In addition, PRDX2 is also the part of inflammatory cascade and can induce TNF-*α* release. In classical inflammatory response, cytokines are released but PRDX2 does not affect mRNA or protein synthesis mediated by liposaccharide (LPS) although it continuously exists in macrophages but in lowered level when stimulated by LPS then released in oxidized form. This study concluded that PRDX2 and thioredoxin (TRX) from macrophages can alter the redox status of cell surface receptors and allow the induction of inflammatory response, providing a potential novel therapeutic target for chronic inflammatory diseases [24].

Overproduction of oxidative stress induces severe cellular damage of the brain in diabetes [29]. Studies documented that higher lipid peroxidation, nitrite levels, malondialdehyde, and total oxidants status were lower in total antioxidant marker enzymes in the brain of diabetic rats [30]. Moreover, studies demonstrated that diabetes induced oxidative stress increases the level of proinflammatory cytokines such as TNF-*α* and interleukin-6 (IL-6) [31] and also upregulates inflammatory molecules like vascular cell adhesion molecule-1 (VCAM-1), intercellular adhesion molecule-1 (ICAM-1), and nuclear factor-kappa B (NF-*κ*B) [31], which leads to degeneration of neurons results in diabetic encephalopathy.

**Figure 1:**
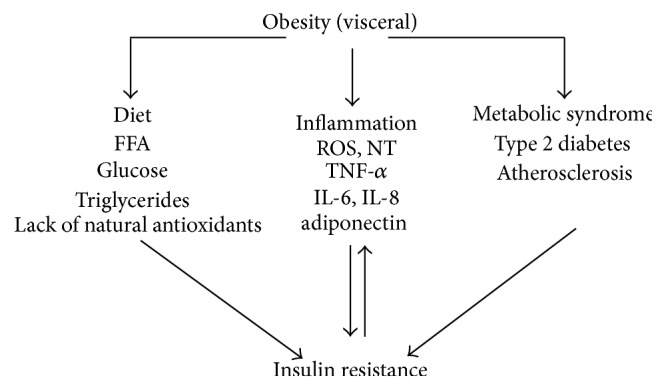
Obesity lifestyle development of chronic diseases through inflammation.

Chronic inflammation is involved in the pathogenesis of several diseases such as insulin resistance, type 2 diabetes mellitus (T2DM), and cardiovascular diseases (CVD); obesity related chronic inflammation factors are described in Figure 1. Inflammation itself cannot be viewed as a disease but should be rather viewed as a biological process. Cotreatment routine significantly decreased the TBARS concentration and DNA fragmentation in the lungs [32].

Study was conducted to test the effect of lemon verbena extract on triglyceride accumulation in the insulin resistant hypertrophic 3T3-L1 cells adipocyte model. Lemon verbena polyphenols decreased the triglyceride accumulation and the ROS generation in hypertrophic adipocytes [33].

## 3. Bioavailability of Polyphenols

Briefly, polyphenols are natural compounds but synthetic and semisynthetic compounds are also available and characterized by the presence of phenolic structural units. Fruits, vegetables, cereals, and beverages are examples of dietary compounds containing polyphenols. More precisely, fruits such as grapes, apple, pear, cherries, and berries and their byproducts contain polyphenols. Moreover, red wine, tea or coffee, chocolates, cereals, and dry legumes also contain polyphenol [34]. Lastly, polyphenols are present in herbs, spices, stems, and flowers. In many countries, polyphenols are daily consumed as part of the diet [35]. Polyphenols are abundant antioxidants in some components of the diet. Daily intake of these dietary compounds may be as high as 1 g/d. Thus, polyphenols are generally higher in food than all other phytochemicals dietary antioxidants [36].

Polyphenols are the secondary metabolites of plants involved in defensive system by including protection from ultraviolet radiation and pathogens [37]. Polyphenols are characterized by bitterness, astringent color, odor, and protection against oxidative processes. To date, more than 8000 phenolic compounds have been identified in the plants. Examples of polyphenols are flavonoids such as flavonols, flavones, isoflavones, anthocyanidins, resveratrol, curcumin, tannins, lignans, and phenolic acids [38]. Polyphenols have some anti-inflammatory and antibiotic properties and may in addition activate the transcription factor Nrf2. Nrf2 plays a key role in cellular protection against oxidative stress and inflammation [39].

Environmental factors have major effect on the dietary polyphenol contents. These factors are type of the soil, exposure of the light, rainfall, culture methods, and fruit yield per tree. Flavonoid concentrations also depend on light exposure. Ripening process decreases or increases the concentration of some phenolic acids [40]. Bioavailability depends on numerous parameters including digestion, absorption, and metabolism. There are no simple relations between quantity of polyphenol and its bioavailability in human diet. Most polyphenols are present in the form of esters [41]. Polyphenols are poorly absorbed from intestine; polyphenols are firstly hydrolyzed by intestinal enzymes or by colonic microflora and then are absorbed. After being modified in different metabolic pathways, they finally come in the blood but not in the initial biological form [41]. Due to its incomplete absorption, polyphenols may reach the colon, where they are metabolized by the intestinal microbiota giving rise to several bacterial metabolites. Micromolar amounts of flavonoids and monophenols are recovered in the feces [42].

## 4. Polyphenols on Oxidative Stress

### 4.1. Antioxidant Properties of Polyphenols

The excessive production of ROS may cause tissue injury that may lead to the inflammatory process [43]. Polyphenol antioxidant activity depends on the structure of their functional groups. The number of hydroxyl groups greatly influences several mechanisms of antioxidant activity such as scavenging radicals and metal ion chelation ability [44]. Polyphenol antioxidant activities are related to their capacity to scavenge a wide range of ROS. Indeed, the mechanisms involved in the antioxidant capacity of polyphenols include suppression of ROS formation by either inhibition of enzymes involved in their production, scavenging of ROS, or upregulation or protection of antioxidant defenses [45].

Polyphenols may reduce the catalytic activity of enzymes involved in ROS generation. Polyphenols are able to protect against oxidative damage through various mechanisms [46]. ROS formation has been reported to enhance free metal ions by reduction of hydrogen peroxidase with generation of the highly reactive hydroxyl radical. Lower redox potentials of the polyphenols are thermodynamically able to reduce highly oxidizing free radicals because of their capacity to chelate metal ions (irons, copper, etc.) and free radical [47]. For instance, quercetin has iron chelating and iron-stabilizing properties.

### 4.2. Interaction of Free Radicals with Polyphenols

Polyphenols may react in plasma membrane with nonpolar compounds present in the hydrophobic inner membrane layer; such changes in the membrane may affect oxidation rate of lipid or proteins. Some flavonoids in the hydrophobic core of membrane may prevent access of oxidants and protect the structure and function of membrane [48]. These processes may help to understand the basic mechanisms of action of polyphenols including cellular interaction and signal transduction.

Interaction of polyphenols with nitric oxide synthases (NOS) activity may modulate the NO production. Xanthine oxidase (XO) is considered as a key source of free radicals, and some flavonoids such as quercetin, silibin, and luteolin have been shown to inhibit such activity. Flavonoids may also reduce the activity of peroxidase and may inhibit the release of free radicals by neutrophils and activation of these cells by *α*1-antitrypsin [49].

### 4.3. Inhibition of Enzymes Involved in Oxidation

**Figure 2:**
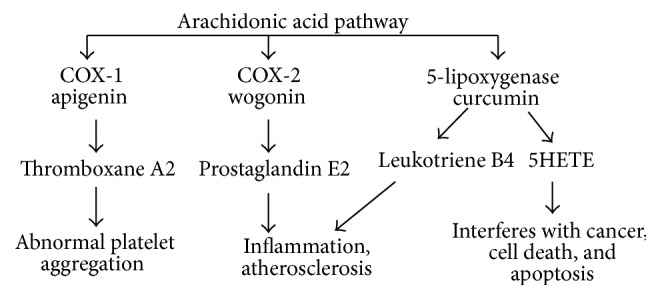
Metabolic pathways involved in arachidonic acid metabolism leading to inflammatory diseases.

Various investigations have shown that different polyphenols modulate the activity of arachidonic acid metabolizing enzymes such as cyclooxygenase (COX), lipoxygenase (LOX), and NOS [50]. Inhibition of these enzymes reduces the production of AA, prostaglandins, leukotrienes, and NO which are among the key mediators of inflammation. Arachidonic acid pathway of inflammation is shown in Figure 2.

Bacterial endotoxins and inflammatory cytokines may stimulate macrophages with resulting increased iNOS expression and NO production and subsequent oxidative injury. Polyphenols may inhibit LPS-induced iNOS gene expression and its associated activity in cultured macrophages [51], thus resulting in decreased oxidative damage.

COX and LOX are the enzymatic activities responsible for the production of metabolites with capacity to increase the oxidative lesion in tissues. Some polyphenols have properties to inhibit the activities of COX and LOX [52]. Oxidative injury to the tissues may be worsened by metabolites notably those produced in the XO pathway. Xanthine dehydrogenase (XDH) activity may convert into XO activity during the ischemia, resulting in the production of ROS. Deceased oxidative injury has been reported with polyphenols thus reducing the activity of XO [53].

## 5. Polyphenols on Inflammation

### 5.1. Modulatory Functions of Polyphenols towards Cells Involved in the Inflammatory Process

Anti-inflammatory activities of the polyphenols such as quercetin, rutin, morin, hesperetin, and hesperidin have been reported in acute and chronic inflammation in animal models (Table 1). Rutin is only effective in the chronic inflammatory processes especially in arthritis; and flavanones are also effective in neurogenic inflammation induced by xylene [54]. Quercetin has been reported to reduce paw edema induced by carrageenan. Paradkar et al., 2004 [55], reported that inflammatory reaction induced by LPS injection can be modulated with daidzin, glycitin, genistein, and their glucosides.

Polyphenols may affect enzymatic and signaling systems which are involved in the inflammatory processes, such as tyrosine and serine-threonine protein kinases. These enzymes are known to be involved in cell activation processes such as T cell proliferation, B lymphocyte activation [56], or cytokine production by stimulated monocytes. Genistein has been reported as a specific inhibitor for tyrosine protein kinase [57]. This latter compound may be involved in some of anti-inflammatory effects, since T cell proliferation is accompanied by phosphorylation of tyrosine of particular proteins. Polyphenols also exhibit an effect on secretory processes of inflammatory cells. Indeed, compounds such as luteolin, kaempferol, apigenin, or quercetin have been documented to represent powerful inhibitors of b-glucuronidase and lysozyme released from neutrophils. These polyphenols in addition significantly inhibit arachidonic acid release from cell membranes [58].

### 5.2. Mechanism of Anti-Inflammatory Effects of Polyphenols

Polyphenols may exert anti-inflammatory effects notably through radical scavenging activities, regulation of cellular activities in inflammatory cells, and modulation of the activities of enzymes involved in arachidonic acid metabolism (phospholipase A2, COX) and arginine metabolism (NOS), as well as the modulation of the production of other proinflammatory molecules.

Molecular mechanisms of polyphenol anti-inflammatory activities include inhibition of enzymes associated with proinflammatory properties such as COX-2, LOX, and iNOS, inhibition of NF-*κ*B and the activating protein-1 (AP-1), activation of phase-II antioxidant detoxifying enzymes, and activation of mitogen activated protein kinase (MAPK), protein kinase-C, and nuclear factor erythroid 2-related factor [59].

Strong evidences are originating from experiments with natural phytochemicals which have been shown to modulate different inflammatory mediators such as metabolites derived from arachidonic acid, various peptides, excitatory amino acids, and cytokines. Furthermore, activities of some second messengers (cGMP, cAMP, protein kinases, and calcium), some transcription factors (AP-1, NF-*κ*B, and protooncogenes), and some enzymes and compounds (iNOS, COX-2), cytokines (IL-1*β*, TNF-*α*), neuropeptides, and proteases [22] are known to be central in the process of inflammation.

## 6. The Roles of Polyphenols in Inflammation-Mediated Chronic Disorder

### 6.1. Polyphenols and Cardiovascular Disease

Antioxidant properties of the polyphenols may be beneficial in the process of inflammation and in inflammation induced carcinogenesis [60, 61]. Many epidemiological and human studies have proposed that daily intake of polyphenols rich diet such as fruits, vegetables, cocoa, tea, and wine may exhibit fruitful effects in humans [62, 63]. Moreover, the meta-analysis studies suggested that higher intake of three cups of tea per day suppress risks by 11% [64] whereas adequate intake of red wine is related to 32% lower risk of cardiovascular disease (CVD) [65]. Although the active role of flavonoids in connection with CVD needs to be debated, in fact, systematic review on soy and cocoa flavonoids exerts health beneficial effects on preventing the risk of CVD [66]. The mechanism of polyphenols on vascular function depends on the ability of nitric oxide synthase (eNOS) and its bioavailability to the endothelium [67, 68]. This vascular nitric oxide regularity mechanism is believed to have involvement of polyphenols with kinase molecular signaling like PI3-kinase/Akt pathway and intracellular Ca^2+^ on eNOS phosphorylation which ultimately results in NO production [69, 70]. Flavanols and flavonols also can interact and lower the occurrences of age-related vascular injury [71] with interaction of MAPK signaling [72] and downregulation of transcription factors (i.e., NF-kB) which causes the reduction in nicotinamide adenine dinucleotide phosphate (NADPH) oxidase [73].

### 6.2. Polyphenols and Neurological Diseases

The dietary modulation of neurological diseases with polyphenols has been reported widely [74, 75]. In addition, daily consumption of high flavonoids diet/beverages can reduce 50% lowering the incidences of dementia [76] and aging [77] and may possibly delay the onset of Alzheimer's disease [78] and also reduce the occurrences of Parkinson's disease [79]. The protective mechanism of polyphenols on neurodegenerative diseases is to modulate neuronal and glial signaling pathways [80]. Polyphenols may downregulate transcription factors NF-*κ*B [81, 82], which react to p38 signaling and are responsible for iNOS induction [83]. It indicates that there might be a link in signaling pathways and transcription factors and cytokine production in exploring the neuroinflammation response in central nervous system. Furthermore, the ability of polyphenols on neuronal signaling exerts the defense in response to neurotoxicity induced by advanced glycation end products (AGEs) [84].

### 6.3. Polyphenols as an Alternative Target for Cancer Therapy

The literature revealed that intuitional supplementation of polyphenols decreases the incidences of different forms of cancer [85]. Polyphenols exert protection against gastrointestinal tract cancers whereas variety of polyphenols in fruits and vegetables are responsible for preventing the incidences of colon cancer [86]. Intake of green tea has been suggested to lower the incidences of biliary tract [87], bladder [88], breast [89], and colon cancer, respectively [90]. Polyphenols may possess anticancer activities by several mechanisms, comprising the elimination of carcinogenic agents [91], modifying cancer cell signaling [92] and cell cycle progression [93], progression of apoptosis [94], and modulation of enzymatic activities [95]. Polyphenols have shown antioxidant properties in tea, red wine, and cocoa, fruit, juices, and olive oil which suppresses cancer formation and development [96], such as interaction with reactive intermediates [97], and stimulates carcinogenesis and mutagens [98]. The compounds in green tea especially flavanol, epigallocatechin gallate (EGCG), possess anticancer properties via apoptotic induction and prevent cell growth by interference with cell cycle regulatory proteins and signaling proteins which are involved in cell proliferation, transformation, and metastasis [92].

## 7. Conclusion and Perspectives

Polyphenols are compounds with various potential biological properties such as antioxidants, anti-inflammatory, antineoplastic, antiaging, cardioprotective, anticancer, and antimicrobial properties. Polyphenols are gaining interest due to their wide applications in different pathological situations.

Oxidative stress activates a variety of inflammatory mediators involved in several chronic diseases. Clinical evidence suggests that oxidative stress and inflammation linked to overproduction of ROS are likely to represent an important component for the development of several diseases including inflammation-associated chronic diseases. Numerous studies performed with animal and cell models suggest that polyphenol dietary intake may be beneficial as adjuvant treatment for the prevention and treatment among such diseases. However, only few clinical studies, notably those done in a double-blind manner, have been performed in order to establish the relevance of these experimental studies for extrapolation to human beings.

A better clarification and understanding of the mechanisms presumably involved in the protective role of polyphenols in adverse situations will help to more precisely define the clinical situations where polyphenol consumption will prove to be beneficial. Such investigation may in addition prove to be useful for the development of new compounds with anti-inflammatory effects.

## Figures and Tables

**Table 1:** Anti-inflammatory activities of some polyphenolic compounds.

Phenolic compounds	Anti-inflammatory activities	Inflammatory markers	Mechanism	References
Apigenin	Inhibiting LPS-induced inflammation	Casp-1, IL-1*β*	Inhibiting NLRP3 inflammasome activation	Zhang et al., 2014 [8]
Catechin	Against monosodium urate-induced inflammation	IL-1*β*	Modulating NLRP3 inflammasome activation	Jhang et al., 2015 [9]
Epigallocatechin gallate	Suppressing melanoma growth	NLRP1, IL-1*β*, NF*κ*B	Inhibiting NLRP3 activation	Ellis et al., 2011 [10]
Epigallocatechin gallate	Protecting endothelial cells against PCB 126 induced inflammation	GST, NQO1, MCP-1, ICAM-1	Modulating AhR/Nrf2 pathway	Han et al., 2012 [11]
Ellagic acid	Ameliorating monocrotaline-induced pulmonary artery hypertension	IL-1*β*, IL-2, IL-4, IL- 6, IL-10, IFN-*γ*, MIP-1, MDA, NLPR3	Suppressing NLRP3 inflammasome activation	Tang et al., 2015 [12]
Green tea	Decreasing PCB 126 induced oxidative stress	SOD1, GSR, NQO1, GST	Stimulating AhR/Nrf2 pathway	Newsome et al., 2014 [13]
Homoplantaginin	Inhibiting palmitic acid-induced inflammation	IL-1*β*, ICAM-1, MCP-1, Casp-1	Interacting with ROS sensitive thioredoxin protein	He et al., 2016 [14]
Luteoloside	Inhibiting proliferation, invasion, and metastasis of HCC cells	ROS, NLPR3, Casp- 1, IL-1*β*	Suppressing NLRP3 inflammasome activation	Fan et al., 2014 [15]
Plant polyphenols	Modulating the inflammatory response of human keratinocytes	MCP-1, IL-10, IL-8, TNF-*α*, IL-6	Impairing phosphorylation EGF induced NF*κ*B via regulating AhR signaling	Potapovich et al., 2011 [16]
Quercetin and allopurinol	Repair of kidney injury	IL-1*β*, IL-18	Suppressing NLRP3 inflammasomes	Wang et al., 2012 [17]
Resveratrol	Ameliorating hepatic metaflammation	IL-1, TNF-*α*, IL-6	Inhibiting NLRP3 activation	Yang and Lim 2014 [18]
Rutin	Reducing inflammation in pancreas	Casp-1, IL-1*β*, ASC- NLRP3, IL-18, TNF-*α*	Suppressing NLRP3 inflammasome activation	Aruna et al., 2014 [19]
Tangeretin	Attenuating oxidative stress and protecting hepatocellular architecture	CYP1B1, CYP2E1, g-GCS, NQO1, HO-1, IL-6, TNF-*α*, IL-1*β*	Stimulating AhR/Nrf2-Keap1 pathway	Arivazhagan and Subramanian 2015 [20]

LPS: lipopolysaccharide; Casp-1: caspase-1; IL: interleukin; NLRP: NOD-like receptor protein; NF-*κ*B: nuclear factor-kappa B; GST: glutathione S-transferase; NQO1 (NAD(P)H: quinone oxidoreductase l); MCP-1: monocyte chemotactic protein-1; ICAM-1: intercellular adhesion molecule-1; AhR: aryl hydrocarbon receptor; IFN-*γ*: interferon-gamma; MIP-1: macrophage inflammatory protein-1; MDA: malondialdehyde; PCB: polychlorinated biphenyls; SOD: superoxide dismutase; GSR: glutathione S-reductase; GST: glutathione S-transferase; HCC: hepatocellular carcinoma cells; ROS: oxygen reactive species; TNF-*α*: tumor necrosis factor-alpha; EGF: epidermal growth factor; ASC: apoptosis associated speck-like CARD containing protein; CYPs, cytochrome P450 dependent monooxygenases; g-GCS: glutamate-cysteine ligase; HO-1: heme oxygenase-1.
